# Imbalance of the Nerve Growth Factor and Its Precursor: Implication in Diabetic Retinopathy

**DOI:** 10.4172/2155-9570.1000483

**Published:** 2015-10-25

**Authors:** Riyaz Mohamed, Azza B El-Remessy

**Affiliations:** 1 Program in Clinical and Experimental Therapeutics, College of Pharmacy, University of Georgia, USA; 2 Culver Vision Discovery Institute, Georgia Regents University, USA; 3 Charlie Norwood Veterans Affairs Medical Center, Augusta, Georgia, USA

**Keywords:** Neurotrophin, Nerve growth factor, Diabetic retinopathy, Acellular capillary, Apoptosis, Neurodegeneration, Inflammation

## Abstract

Diabetic retinopathy is the leading cause of blindness in working age in US and worldwide. Neurotrophins including nerve growth factor (NGF), brain-derived neurotrophic factor (BDNF), neurotrophin-3 (NT-3) and neurotrophin-4 (NT-4) are known to be essential for growth, differentiation and survival of neurons in the developing and mature retina. Nevertheless, a growing body of evidence supports an emerging role of neurotrophins in retinal diseases and in particular, diabetic retinopathy. Neurotrophins are initially synthesized in a pro-form and undergo proteolytic cleavage to produce the mature form that activates two distinctive receptors, the tyrosine kinase tropomycin receptor (Trk) and, to lesser extent, the common low affinity p75 neurotrophin receptor (p75^NTR^). Despite tight glycemic and metabolic control, many diabetic patients continue to experience progressive retinal damage. Understanding the molecular events involved in diabetic retinopathy is extremely important to identify novel therapeutic strategies to halt the disease progression. Diabetes induces imbalance in neurotrophins by increasing its proform, which is associated with upregulation of the p75^NTR^ receptor in the retina. A growing body of evidence supports a link between the imbalance of pro-neurotrophins and early retinal inflammation, neuro-and microvascular degeneration. Therefore, examining changes in the levels of neurotrophins and its receptors might provide a therapeutically beneficial target to combat disease progression in diabetic patients. This commentary aims to highlight the impact of diabetes-impaired balance of neurotrophins and in particular, the NGF and its receptors; TrkA and p75^NTR^ in the pathology of DR.

## Diabetic Retinopathy

Diabetic retinopathy (DR) is a severe sight threatening complication of diabetes mellitus and the leading cause of blindness in the worldwide. The retina is a typical neurovascular system with a delicate organization of neurons, glia cells and blood vessels. Although DR was previously perceived as a sole microvascular complication, it is now widely accepted that diabetes affects multiple cell types in the retina. The major mechanisms of DR pathogenesis appeared to be retinal neurodegeneration, inflammation, alteration of microvasculature including barrier dysfunction, loss of pericyte and development of acellular capillaries that eventually cause ischemia and hypoxia [1,2]. To meet the oxygen demands, retinal neovascularization is triggered in response to several proangiogenic factors including vascular endothelial growth factor (VEGF) leading to abnormal growth of new leaky blood vessels [1,2]. The inner blood-retina barrier (BRB) is located within endothelium of capillaries and interconnected by the processes of glia (astrocytes and Müller cells) as well as patches of pericytes [3]. The tight BRB serves essential role in regulating the microenvironment and preserving neuronal function. Early breakdown of BRB as well as leaky blood vessels arising from late neovascularization can cause macular edema and ultimately vision loss [4]. Current treatments like photocoagulation, vitrectomy and anti-VEGF therapy are effective, yet limited with considerable side effect [5,6]. Understanding the molecular events that govern DR progression is critical to devise new therapeutic strategies for treatment. Earlier work by Hammes et al. 1995 showed that treatment of diabetic rats with nerve growth factor (NGF) prevented early retinal ganglion death, Muller cell activation and development of acellular occluded capillaries [7], suggesting the involvement of NGF and other neurotrophins in pathophysiology of DR. In the next sections, we will highlight the findings of NGF and its receptor recently identified in experimental models and clinical samples of DR.

## Neurotrophin and Receptor System

Neurotrophins (NTs) are secreted growth factors that regulate neuronal differentiation, survival, neurite outgrowth, synaptic formation, and plasticity [8]. There are four types of neurotrophins that have been characterized in mammals including NGF, Brain-derived neurotrophic factor (BDNF), neurotrophin-3 (NT-3) and neurotrophin-4 (NT-4) [9-11]. They are collectively called neurotrophins, since; they are derived from a common gene, with similar sequence and structure. All neurotrophins are initially synthesized in a pro-form, which later undergoes proteolytic cleavage intracellularly by plasmin and furin and extracellularly by matrix metalloproteinase to produce the mature form of neurotrophins [8,12-14]. All mature neurotrophins exert their action by interacting with two distinctive receptors, the high affinity tyrosine kinase tropomycin receptor (Trk) and the common low affinity p75 neurotrophin receptor (p75^NTR^). NGF will preferentially bind to its receptor TrkA and BDNF and NT4 bind TrkB receptor with high affinity whereas NT-3 has the binding affinity toward TrkC receptor. The mature neurotrophin for example, NGF binds to its respective TrkA receptor resulting in receptor auto-phosphorylation and initiation of different signaling pathways like PI3 kinase, Ras/Extracellular signal-regulated kinases (ERK), Akt 1, protein kinase C (PKC) pathway to promote neuronal survival, differentiation and cell growth [15,16]. The affinity of Trk-receptors for mature neurotrophin is enhanced by the association of Trk with p75^NTR^ receptors [17].

Similar to the mature neurotrophin, pro-form of a neurotrophin (proNT) can display its independent biological activity by binding p75^NTR^ and interacting with sortilin, a member of VPS-10p domain receptor family to elicit cell death [12,18,19]. The p75^NTR^ belongs to tumor necrosis factor (TNF) receptor superfamily and thus its activation during cell death signal involves activation of TNF receptor associated factors (TRAFs), nuclear Kappa B (NF_k_B) and ceramide [20-22]. The p75^NTR^ has ability to bind both mature and pro-form of neurotrophins to interact with other adaptor proteins to produce neuronal growth, proliferation or cell death [16,18,2,23]. Activated p75^NTR^ receptor undergoes intramembrane proteolysis by sequential α-secretase and γ-secretase-catalyzed cleavage of extracellular and intracellular domain respectively to release p75^ICD^. Interestingly, p75^NTR^ receptor lacks the catalytic domain and signaling proceeds through ligand-induced recruitment and association of an effector molecules with p75^ICD^ [17,22,24]. The p75^ICD^ can then interact with proteins in the cytoplasm or to be translocated to nucleus where it may directly regulate transcription [25-29]. Initially, it was assumed that neuronal cells express and utilize neurotrophins, but subsequent studies showed that other non-neuronal cell types also express neurotrophins [30-32].

## Nerve growth factor (NGF)

NGF is a pleotropic factor that extends its biological activity from central and peripheral nerve system to the immune, endocrine and visual system [9,33]. The duration and magnitude of NGF receptor signaling is dependent on the distribution ratio of TrkA and p75^NTR^ receptor on the cell surface [34]. Physiologically, NGF and TrkA are expressed in the anterior segment of the eye like iris, ciliary body, lens, cornea and conjunctiva and NGF is released into aqueous humor [30,35,36]. In the retina, NGF is produced and utilized by retinal ganglion cell (RGCs) and glial cells in a paracrine and autocrine fashion [37,38]. The neurotrophin receptor TrkA is expressed in RGC, glial cell and endothelial cell whereas the low affinity p75^NTR^ receptor is expressed widely across the retina, mainly within the Muller cells and to less extent in RGCs [39], pericytes [40], endothelial cells [41], retinal pigment epithelium [42] and photoreceptor cells [43]. TrkA and p75^NTR^ are also expressed in most intra-ocular tissues, including lens, vitreous, choroid, iris, and trabecular meshwork [30] and in immune cell [44]. Neurotrophins are not only involved in regulation of retinal development but also plays a key role in regeneration of neuronal circuits in the visual system during retinal injury or retinal degenerative disease [9].

**Figure1:**
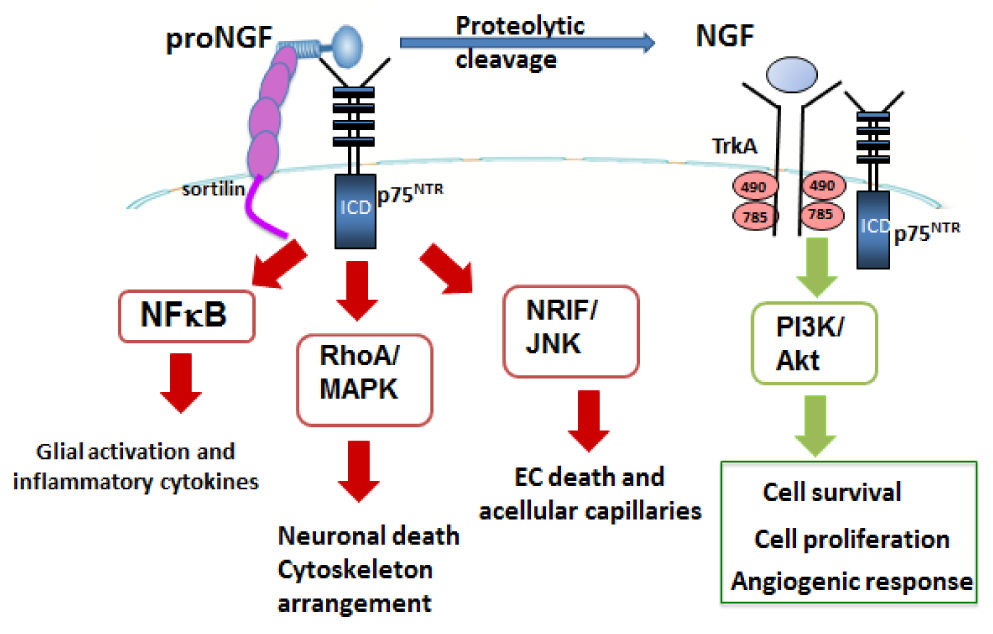
Schematic representation of the multiple pathways by which proNGF/NGF can contribute to diabetic retinopathy. Diabetes impairs the homeostasis of NGF by decreasing the proteolytic cleavage of the proform resulting in increasing proNGF levels and decreasing NGF levels. Mature NGF binds to tyrosine kinase TrkA receptor in combination with p75^NTR^ causing autophosphorylation of TrkA receptor and activation of P13K/Akt pathway leading to cell proliferation, cell survival and angiogenic response. ProNGF preferentially binds to p75^NTR^, in combination with its co-receptor sortilin, to activate multiple pathways depending on the interaction of the intracellular domain (ICD) and a given adaptor protein. Interaction of p75^NTR^ ICD with NFkB results in activation of proinflammatory cytokine production. Interaction of ICD with RhoA/MAPK pathway resulting in neuronal death, cytoskeleton arrangement and BRB breakdown. Interaction of the ICD with the neurotrophin interacting factor (NRIF) will activate c-Jun kinase (JNK) resulting in endothelial cell (EC) apoptosis and formation of acellular capillaries, surrogate marker of ischemia.

Alternation in NGF levels has been correlated with various diabetic microvascular complications including retinopathy, nephropathy, and neuropathy. As shown in Table 1, prior studies showed that serum and tear NGF levels were higher in DR patients and correlated well with HbA1c, severity of hyperglycemia, progression of the disease, and the existence of diabetic nephropathy [45]. Similarly, diabetes increased serum and kidney levels of NGF in an experimental model of diabetic nephropathy [46]. Another independent study reported significant increases in serum NGF level in patients with diabetic neuropathy [47]. However, the expression of NT-3 and NT-4 was upregulated in vitreous fluid in PDR patients [11] Figure 1.

## Imbalance of neurotrophins and their receptors in the diabetic retina

In the above mentioned studies, levels of NGF were detected using ELISA or mRNA expression techniques, which could not distinguish between the precursor and mature NGF form. With availability of better tools and antibodies that recognize proNGF apart from NGF, our group had discovered that maturation of proNGF is impaired in the diabetic milieu, resulting in increased proNGF expression and decreased NGF expression. This observation of increased proNGF and decreased NGF was confirmed in experimental diabetic retina and isolated retinal Muller cell (rMC-1) cultured in high glucose [48,49]. Furthermore, this increased proNGF/NGF imbalance was demonstrated in vitreous fluids of diabetic patients as well as in aqueous humor samples from PDR patients compared to non-diabetic patients [49]. Diabetes-induced proNGF/NGF imbalance was attributed to the reduction in expression and activity of MMP-7, the enzyme that cleaves proNGF to form mature NGF [12,49] resulting in accumulation of proNGF and decreases in NGF levels in the retina [49]. In parallel, our recent study [50] showed that diabetes-induced imbalance of proNGF/NGF observed in aqueous humor fluid was mirrored in the serum of the same PDR patients (Table-1). This interesting finding highlights the possible contribution of the proNGF/NGF imbalance as a biomarker for DR but does not exclude the possibility that imbalance can also contribute to the pathogenesis of the disease. As shown in table-1, recent work showed that significant reductions in BDNF level in vitreous and serum samples of PDR patients compared to non-diabetic patients [51]. Similar decreases were observed in serum of PDR patients as well as in diabetic rat retina that coincided with decreased retinal TrkB expression [52].

The imbalance in proNGF/NGF during diabetes was associated with alteration and expression of TrkA and p75^NTR^ receptors. While diabetes did not alter TrkA levels, the activation of the receptor was significantly decreased in human and diabetic rat retina [37,53]. The impaired TrkA activity was associated with upregulation of p75^NTR^ receptor, resulting in favoring activation of cell death pathway and neurodegeneration in the diabetic retina [37,49]. Interestingly, even in a non-diabetic milieu, overexpression of the cleavage-resistance proNGF plasmid significantly reduced NGF level and increased p75^NTR^ expression in rat retina [41,54,55]. Another study demonstrated that the imbalance of proNT3/NT3 was associated with upregulation of p75^NTR^ expression, leading to photoreceptor degeneration in a selective Muller cell ablation model [43]. Together, these observations suggest that alteration of proneurotrophin to the mature neurotrophin ratio coincided with upregulation of p75^NTR^ expression and resulted in shifting the homeostasis toward cell death signals. In the next sections, we will examine evidence from literature on how imbalance in proNGF/NGF ratio correlates with early characteristics of diabetic retinopathy including: retinal inflammation, neuro- and microvascular degeneration.

## Impact of proNGF/NGF imbalance on retinal inflammation

Retinal inflammation is recognized as important factor in the pathogenesis of a wide array of retinal diseases including DR [56-58]. During diabetes, the imbalance of proNGF/NGF expression favors proNGF/p75^NTR^ pathway, which has been linked to retinal inflammation [48,49], and apoptosis [37,55]. Diabetes and proNGF overexpressing model showed selective increases in proNGF and p75^NTR^ within the Muller cells with increased glial fibrillary acidic protein, a sign of glial cell activation [38,48,49,59,60]. These findings signify the importance of p75^NTR^ in regulation of inflammatory mediators and their downstream signaling. Activation of proNGF/p75^NTR^-mediated release of inflammatory mediators and glial cell activation can also contribute to pathogenesis of DR. Overexpression of proNGF-independent of diabetic milieu-induces significant expression of p75^NTR^, NF-kB, and inflammatory mediators TNF-α, IL-1β in Muller cells [55,59], but not in retinal endothelial cell [41]. We and others have shown that p75^NTR^ activates NF_k_B under stressed condition [6, 62]. The underlying mechanism of how p75NTR can activate NF_k_B is not fully understood. One possibility is that activation of p75^NTR^ causes intramembranous proteolysis to liberate p75^ICD^, which can recruit intracellular effector proteins like TRAF6 [63] or DNA binding protein neurotrophin interacting factor (NRIF) to activate NF_k_B [41].

Deletion or inhibition of p75^NTR^ receptor in Muller cells blunted the proNGF-mediated pro-inflammatory response [59]. Similarly, genetic deletion of p75^NTR^ reversed diabetes-induced imbalance of proNGF/NGF by decreasing proNGF and restoring NGF levels. These effects were associated with decreases in inflammatory mediators including NFkB, pNFkB and TNF-α and RGC death, glial cell activation and vascular leakage [59]. In control non-diabetic animals, deletion of p75^NTR^ causes increases in basal expression of proNGF and the membrane bound TNF-α protein level, but not in their mRNA level. These observations suggest additional role for p75^NTR^ in proNGF and TNF-α protein processing via posttranslational modification rather than regulation of transcription in the non-diabetic condition.

## Impact of proNGF/NGF imbalance on retinal neuronal death

Recent work demonstrated the impact of proNGF\NGF imbalance on neurodegeneration using transgenic mice that overexpress proNGF. In this model, proNGF overexpression triggered neurodegeneration and learning and memory deficits [64]. Although NGF plays important role in survival and death during retinal neurogenesis, the role of NGF in the diabetic retina was not fully understood. Under pathological condition proNGF or proBDNF is secreted and acts as distinct ligand to promote neuronal apoptosis by directly binding to p75^NTR^ receptor and transmembrane receptor sortilin [12,16,19,20]. Diabetes and proNGF overexpression triggered neurodegeneration that was associated with increased expression of p75^NTR^ and apoptosis marker including caspase-3 and cleaved poly (ADP-ribose) polymerase [49,55]. These effects were accompanied by increased activation of Rho kinase, p38MAPK and JNK leading to apoptosis in primary RGC culture and in diabetic retinas [49, 55]. Another mechanism by which proNGF can induce RGC death is through paracrine effect of proNGF/p75^NTR^-mediated secretion of TNF-α by Muller glial cells [59,65]. Recent work showed that NGF supplementation reduced RGC death in diabetes and glaucoma rat model [7,66,67]. Together, these studies support the notion that restoring NGF level is neuroprotective in diabetic retina.

## Impact of proNGF/NGF imbalance on retinal vascular permeability

During diabetes, retinal vasculature becomes leaky, leading to increased albumin flux into the retina, fluid accumulation, and macular edema that may progress to vision loss [68]. Earlier work showed that diabetes-induced breakdown of the blood retina barrier was associated with increases in proNGF and decreases in NGF [49]. Overexpression of the cleavage-resistance proNGF increased vascular permeability in heathy rat retinas [54]. Deletion of p75^NTR^ receptor prevented the diabetes-induced blood retinal barrier breakdown in diabetic mice [59]. Further, in rMC-1 cell and in diabetic mouse retina, proNGF induced secretion of inflammatory mediators including TNF-α, which is known to induce vascular permeability and endothelial cell death [69-71]. However, pharmacological inhibition of p75^NTR^ receptor or its cleavage blocked TNF-α in Muller cells and its mediated vascular permeability. In parallel, genetic deletion of p75^NTR^ prevented diabetes-induced BRB breakdown without significant alteration of VEGF mRNA levels suggesting the importance of p75^NTR^ receptor in mediating vascular permeability during diabetes [38,59]. VEGF is a known regulator of vascular permeability and angiogenesis in ocular diseases including DR. NGF has been shown to stimulate VEGF production in cultured primary astrocytes in vitro and in hind limb ischemia diabetic model [72,73]. Furthermore, intravitreal injection of bevacizumab, an anti-VEGF antibody down-regulated NGF and increased retinal cell apoptosis in rabbits [74]. In contrast, anti-NGF antibody application reduced the level of NGF and enhanced the expression of VEGF in rodent retina [75]. Although many studies reported interaction of NGF and VEGF, but whether proNGF can directly modulate VEGF levels and retinal vasculature remain to be addressed.

## Impact of proNGF/NGF imbalance on retinal vascular cell death

Diabetes-induced vascular cell death causes formation of acellular capillaries, a hall mark of retinal ischemia that triggers the progression of DR from background retinopathy to PDR and blindness [68]. A land mark study by Hammes et al showed that NGF supplementation promoted endothelial cell survival and prevented pericyte loss as well as formation of occluded capillaries in the diabetic retina [7]. Using a non-diabetic model, our recent work showed that overexpression of proNGF increased the ratio of proNGF/NGF and resulted in apoptosis of endothelial cells and significant occluded (acellular) capillaries formation [41]. Overexpression of proNGF reduced NGF and TrkA phosphorylation and activated p75^NTR^ mediated apoptosis by increasing JNK kinase, p38MAPK and cleaved-PARP activity by translocating p75^NTR^ and DNA binding protein, NRIF into the nucleus and form a complex, which is essential for p75^NTR^ mediated apoptotic signals [41]. Silencing p75^NTR^ expression prevented proNGF-induced p75^NTR^ and p75^ICD^ expression and restored the balance by decreasing the proNGF/p75^NTR^ level and increasing NGF/TrkA expression in a rodent retina and in retinal endothelial cells [4,54].

Prior studies showed that hypoxia induced the expression of p75^NTR^ in retinal pigment epithelium, and promoted cell death of vascular smooth muscle and endothelial cells [76,77]. In oxygen induced retinopathy mouse model, NGF contributed to retinal neovascularization by activating TrkA receptor [78]. Whereas, knocking down of p75^NTR^ suppressed hypoxia induced pro-angiogenic factors and promoted the anti-angiogenesis-related factors [42]. These results indicated that p75^NTR^ could be a potential therapeutic target for hypoxia or oxidative stress diseases including DR.

## Restoring the balance of NGF/proNGF as potential therapeutic strategy

Diabetes-induced imbalance of proNGF/NGF resulted in upregulation of proNGF/p75^NTR^ axis and downregulation of NGF/TrkA axis. This imbalance appears to play critical role in early pathogenesis of DR such as retinal neurodegeneration, inflammation and vascular dysfunction that eventually leads to blindness. Restoring balance the between proneurotrophin/mature neurotrophin represents a potential promising therapeutic strategy to overcome retinal degenerative diseases including DR [79].

Treatment with NGF prevents the early retinal cell apoptosis and development of acellular occluded capillaries [7]. However, injection of anti-NGF antibody worsened RGC loss in experimental diabetic rat [80]. Furthermore, administration of NGF eye drops restored TrkA levels in the retina, and protected RGCs from degeneration in experimental diabetic model [66,80] and in glaucoma rat model [81] suggesting that NGF treatment can restore NGF/proNGF balance during retinopathy. Inhibiting oxidative stress and tyrosine nitration using green tea extract, peroxynitrite decomposition catalyst or atorvastatin reduced the burden of proNGF/NGF imbalance in the diabetic retina. Restoring NGF/proNGF balance using these treatments prevented diabetes-induced retinal neurodegeneration [37,48,49] and vascular permeability [49].

Genetic deletion or silencing of p75^NTR^ prevented proNGF accumulation and restored the mature NGF level and maintained the balance between NGF and proNGF to normal level in diabetic retina [59] or in the non-diabetic retina [41]. Genetic deletion or silencing of p75^NTR^ also prevents proNGF-induced retinal inflammation, vascular permeability and retinal neurodegeneration in the diabetic retina [59] or development of acellular capillaries in a non-diabetic model [41]. Furthermore, restoring NT3/proNT3 balance prevented photoreceptor degeneration in a model of selective Muller cell ablation. In the latter study, restoring the balance was achieved using intravitreal injection of mature NT3 or by neutralizing p75^NTR^ using a specific antibody. Therefore, targeting p75^NTR^ expression or activity may provide a “druggable target” for the treatment of retinal degenerative diseases including DR.

In summary, neurotrophins are known to be essential for growth, differentiation and survival growth factors in the developing and mature retina. However, a growing body of evidence supports the evolving and critical role of neurotrophins in retinal diseases and in particular, diabetic retinopathy. Diabetes alters the homeostasis of NGF by favoring accumulation of proNGF at the expense of the mature NGF. It appears that the imbalance of proNGF/NGF is critical for various early endpoints for diabetic retinopathy including retinal inflammation, neurodegeneration, vascular permeability and development of acellular capillaries. Restoring the balance of NGF/proNGF and targeting proNGF/p75^NTR^ axis may be potential therapeutic strategy to prevent early signs of diabetic retinopathy.

## Figures and Tables

**Table 1:** A summary of studies that determined the level of neurotrophins in the eyes of diabetic patients.

Neurotrophin	Fluid/Tissue	Technique	Reported	Disease state	References
NGF	Serum	ELISA	Increase	DR/PDR	[45]
NGF	Tear	ELISA	Increase	DR/PDR	[45]
NGF	Serum	ELISA	Increase	DN	[47]
NGF	Aqueous humor	Immunoblot	Decrease	PDR	[49,50]
NGF	Vitreous fluid	Immunoblot	Decrease	DR	[49,50]
NGF	Serum	Immunoblot	Decrease	PDR	[50]
proNGF	Aqueous humor	Immunoblot	Increase	PDR	[49,50]
proNGF	Vitreous fluid	Immunoblot	Increase	DR	[49]
proNGF	Serum	Immunoblot	Increase	PDR	[50]
BDNF	Serum	ELISA	Decrease	NPDR, PDR	[51]
BDNF	Vitreous	ELISA	Decrease	NPDR, PDR	[51]
BDNF	Serum	ELISA	Decrease	PDR	[52]
NT3/NT4	Vitreous fluid/Serum	Immunoblot	Increase	PDR	[11]

DN: Diabetic Neuropathy; DR: Diabetic Retinopathy; PDR: Proliferative Diabetic Retinopathy; NPDR: Non-Proliferative Diabetic Retinopathy
